# The Physician’s Visiting List

**Published:** 1894-12

**Authors:** 


THE PHYSICIAN'S VISITING LIST for 1895 (Lindsay & Blackiston's). Forty-fourth year of its publication. Philadelphia: P. Blackiston, Son, & Co., 1012 Walnut Street. Prices: 25 patients a week, $1.00; 50 patients a week, $1.25; 75 patients a week, $1.50; 100 patients a week, $2.00.

This book has been repeatedly noticed in these pages and is the one used by the editor in his practice. The edition for the coming year contains among many other valuable things a list of new remedies; examination of urine; differential diagnosis of Bright's disease; diagnosis of the simpler diseases of the eye; table of eruptive fevers; and a new complete table for calculating the period of utero-gestation.

